# Effect of Glycerol on Fosfomycin Activity against *Escherichia coli*

**DOI:** 10.3390/antibiotics11111612

**Published:** 2022-11-12

**Authors:** Miriam Ortiz-Padilla, Inés Portillo-Calderón, Ana Velázquez-Escudero, Jesús Rodríguez-Baño, Álvaro Pascual, José Manuel Rodríguez-Martínez, Fernando Docobo-Pérez

**Affiliations:** 1 Unidad Clínica de Enfermedades Infecciosas y Microbiología, Hospital Universitario Virgen Macarena, 41009 Sevilla, Spain; 2 Departamento de Microbiología, Facultad de Medicina, Universidad de Sevilla, 41009 Sevilla, Spain; 3 Instituto de Biomedicina de Sevilla IBIS, Hospital Universitario Virgen Macarena/CSIC/Universidad de Sevilla, 41009 Sevilla, Spain; 4 Centro de Investigación Biomédica en Red en Enfermedades Infecciosas (CIBERINFEC), 28029 Madrid, Spain; 5 Departamento de Medicina, Facultad de Medicina, Universidad de Sevilla, 41009 Sevilla, Spain

**Keywords:** fosfomycin, antimicrobial resistance, optimization treatment

## Abstract

Fosfomycin is an antimicrobial that inhibits the biosynthesis of peptidoglycan by entering the bacteria through two channels (UhpT and GlpT). Glycerol is clinically used as a treatment for elevated intracranial pressure and induces the expression of *glpT* in *Escherichia coli.* Glycerol might offer synergistic activity by increasing fosfomycin uptake. The present study evaluates the use of glycerol at physiological concentrations in combination with fosfomycin against a collection of isogenic mutants of fosfomycin-related genes in *E. coli* strains. Induction of fosfomycin transporters, susceptibility tests, interaction assays, and time-kill assays were performed. Our results support the notion that glycerol allows activation of the GlpT transporter, but this induction is delayed over time and is not homogeneous across the bacterial population, leading to contradictory results regarding the enhancement of fosfomycin activity. The susceptibility assays showed an increase in fosfomycin activity with glycerol in the disk diffusion assay but not in the agar dilution or broth microdilution assays. Similarly, in the time-kill assays, the effect of glycerol was absent by the emergence of fosfomycin-resistant subpopulations. In conclusion, glycerol may not be a good candidate for use as an adjuvant with fosfomycin.

## 1. Introduction

Bacterial resistance has been steadily increasing in recent decades, along with the lack of new active drugs, leading to the need to reuse old antimicrobial agents as an alternative strategy [1,2] to combat increased morbidity and mortality worldwide [3]. Fosfomycin is a broad-spectrum antimicrobial approved for the treatment of uncomplicated urinary tract infections, soft tissue infections, and sepsis caused by Enterobacteriaceae [4,5]. The bactericidal action of fosfomycin is achieved by interrupting the first step of peptidoglycan biosynthesis by blocking the MurA enzyme, thus requiring entry into the cytoplasm. This entry is mediated by the GlpT (glycerol-3-phosphate transporter) and UhpT transporters (hexose phosphate transporter), which belong to the major facilitator superfamily [6]. The expression of these transporters is regulated by many factors. As many other genes involved in the catabolism of secondary carbon sources, both are controlled by the concentration of cyclic AMP (cAMP, synthesized by adenylate cyclase encoded by the *cyaA* gene) bound to its transcriptional dual regulator CRP (cAMP receptor protein receptor) [7]. Therefore, the absence of the *cyaA* gene leads to increased resistance to fosfomycin [8]. Moreover, each transporter is induced by its own substrate. UhpT is induced by the presence of G6P, which is detected in the periplasmic space by the two-component system UhpBC, where UhpB phosphorylates UhpA acting as a transcriptional activator of *uhpT*. Thus, mutations in any gene in this system lead to increased resistance to fosfomycin [9]. With regard to GlpT, this transporter is induced by the presence of G3P, which binds to GlpR, a transcriptional repressor of *glpT*, causing a loss of affinity for the promoter, thus inducing its transcription. Loss of function of this gene would increase the sensitivity to fosfomycin [10]. In this sense, the main mechanism of resistance in *E. coli* clinical isolates is the loss of function of these transporters or genes involved in their regulation [6]. Thus, to fully observe fosfomycin activity, glucose-6-phosphate (G6P, inductor of UhpT) is added for fosfomycin susceptibility assays according to the CLSI or EUCAST guidelines. However, the role of the GlpT transporter in susceptibility testing remains unclear. Although the addition of glycerol-3-phosphate induces the GlpT transporter, it also reduces the transport of fosfomycin by occupancy of the transporter site [11]. Alternative activators should be explored to overcome this problem in order to increase the activity of fosfomycin. In this sense, glycerol could be a good candidate for this function. Glycerol is a triol that can be used by *E. coli* as a carbon source and has also been used orally or intravenously in clinical practice as a potent osmotic dehydrating agent in the treatment of elevated intracranial pressure [12]. In *E. coli*, glycerol enters the bacterium through passive diffusion or through the glycerol facilitator, the GlpF transporter [13]. Once inside, it is phosphorylated by GlpK kinase, producing intracellular glycerol-3-phosphate. This endogenous glycerol-3-phosphate can remove the GlpR repressor from the *glpT* promoter region, activating its transcription [13,14,15]. The use of this carbon source could solve the problem of activating the *glpT* transporter without reducing the intake of fosfomycin. However, fosfomycin activity has not been previously studied in combination with glycerol. Therefore, the objective of the present study is to characterize and evaluate the activity of fosfomycin using glycerol at clinically physiological concentrations for the activation of the GlpT transporter.

## 2. Results

### 2.1. Fosfomycin Transporters Promoters Activity

**Figure 1:**
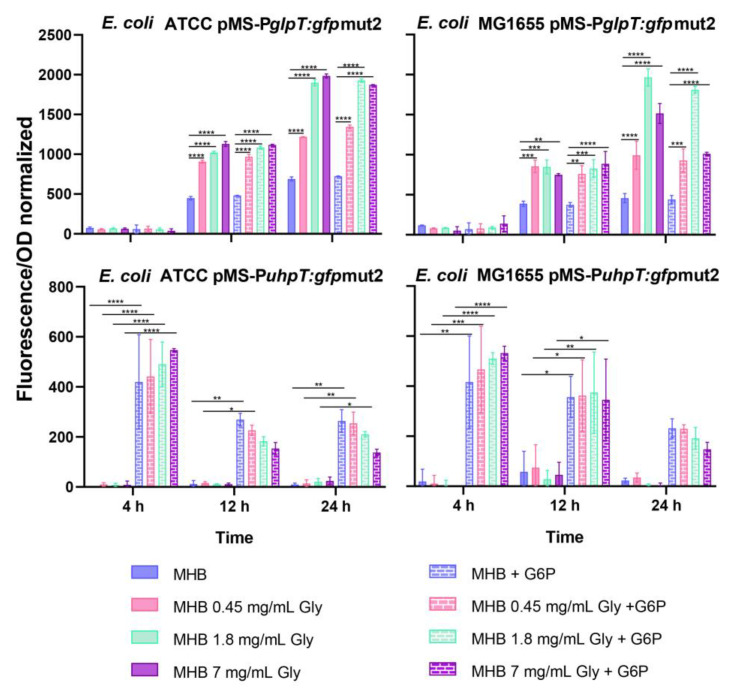
Induction of the *glpT* and *uhpT* genes in the ATCC25922 and MG1655 strains. Fold induction is GFP fluorescence after 4, 12, and 24 h of exposure, normalized to promoterless strains. Error bars represent standard deviation. Significant *p-*values compared to their corresponding (*: *p* < 0.05; **: *p* < 0.01; ***: *p* < 0.001; ****: *p* < 0.0001).

The results on the promoter activity of *glpT* and *uhpT* at the time points 4 h, 12 h, and 24 h are shown in Figure 1. Furthermore, the 24-h fluorescence kinetics is shown in the Supplementary Materials (Figure S1). The presence of glycerol produced a significant increase in *glpT* transcription for both strains at 12 and 24 h. This increase was minimal or absent within the first 4 h, but after this initial lag period, *glpT* transcription increased during the remainder of the assay. In the case of *E. coli* ATCC25922, the highest transcription of *glpT* was observed at the maximum glycerol concentration of 7 mg/mL. While in the case of *E. coli* MG1655, the maximum activity of the *glpT* promoter was observed at a glycerol concentration of 1.8 mg/mL. The addition of G6P did not modify the expression of *glpT.* Regarding the promoter activity of *uhpT,* the addition of G6P, but not glycerol, increased the expression of *uhpT* within the first 4 h, followed by a gradual decline.

**Figure 2:**
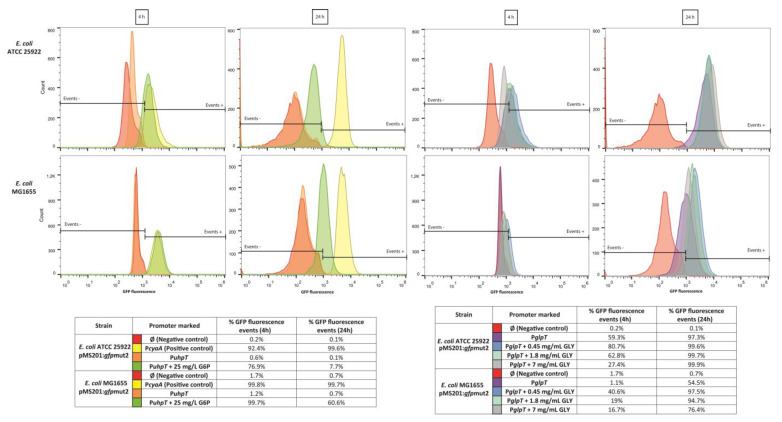
Transcriptional analysis of *glpT, uhpT, and cyaA* expression in *E. coli* ATCC25922 and *E. coli* MG1655 at 4 h and 24 h. GFP fluorescence in *E. coli* cells carrying the transcriptional fusions (P*_glpT_-gfp,* P*_uhpT_-gfp, and* P*_cyaA_-gfp*) and grown in MHB alone or supplemented with glucose-6-phosphate (G6P) or glycerol (GLY). The red and yellow areas identify the regions considered negative and positive for the fluorescence signal, respectively (as assessed with cells carrying the empty pMS201vector or with P_cya_-*gfp* fusion).

The results of flow cytometry are shown in Figure 2. In the absence of glycerol and G6P, no expression of the *uhpT* gene was observed for *E. coli* ATCC25922 and *E. coli* MG1655. The addition of G6P increased the percentage of positive events at 4 h (76.9% and 99.7%). However, the fluorescence decreased to 7.7% and 60% after 24 h. Regarding the expression of the *glpT* gene in MHB alone at 4 h, 59.3% of the *E. coli* ATCC25922 population and 1.1% of the *E. coli* MG1655 population showed *glpT* expression. The addition of glycerol increased the percentage of positive events for both strains, except for the case of *E. coli* ATCC25922 with 7 mg/mL of glycerol. At 24 h, almost all the population of *E. coli* ATCC25922 (97.3%) and half of the population of *E. coli* MG1655 (54.5%) showed fluorescence. The addition of glycerol increased the percentage of positive events for both strains, reaching almost 100% of positive events in *E. coli* ATCC25922 and nearly 95% in *E. coli* MG1655, except with 7 mg/mL of glycerol. The fluorescence intensity was higher in the case of the *E. coli* ATCC25922 strain, closer to the intensity obtained by the positive control (pMS201-P*cyaA*::*gfp*mut2).

### 2.2. Susceptibility Testing

**Figure 3:**
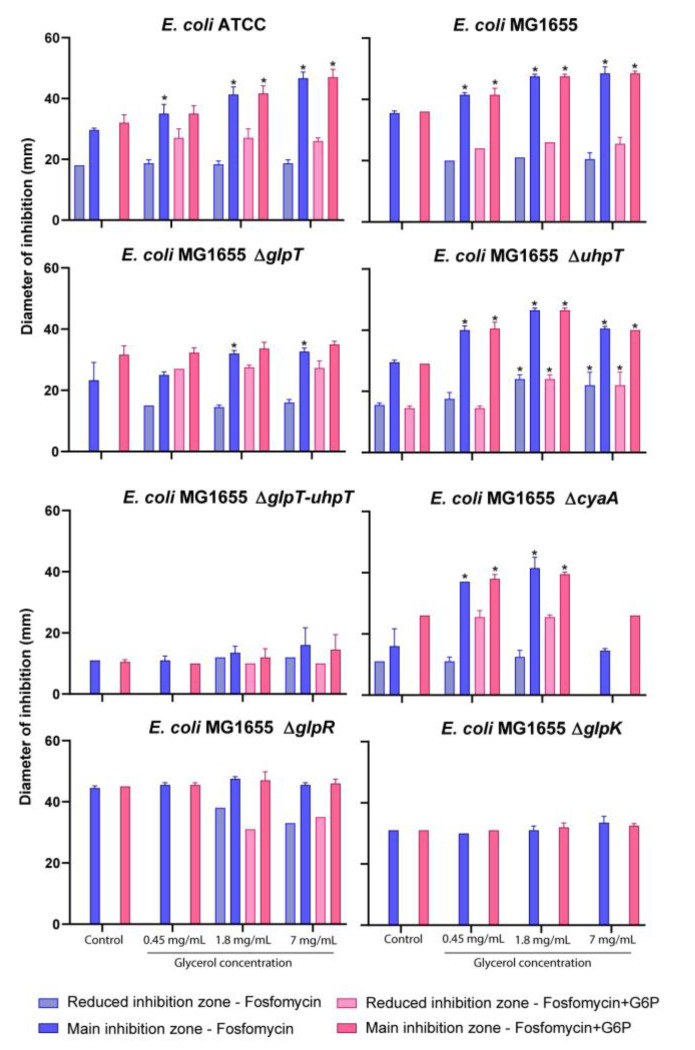
Results of fosfomycin susceptibility by disk diffusion assay with the addition of 0.45, 1.8, and 7 mg/L of glycerol and/or 25 mg/L of glucose-6-phosphate (G6P). The dark-colored columns show the diameter of the main inhibition zone (mean, mm), and the light-colored columns show the diameter of the reduced inhibition zone (mean, mm). Error bars represent the standard deviation. * denotes *p* < 0.05 with respect to their respective control.

The fosfomycin MICs, performed with the reference method, for the isogenic collection are shown in Table 1, and the results of disk diffusion are shown in Figure 3.

With the agar dilution assay, the fosfomycin MIC for *E. coli* ATCC25922 was 2 mg/L with the addition of G6P. The absence of G6P increased the MIC to >64 mg/L, and the addition of 7 mg/mL glycerol did not restore fosfomycin susceptibility. With respect to the *E. coli* MG1655 strain, similar results were observed with a MIC of 2 mg/L with G6P, 64 mg/L without G6P, and 32 mg/L without G6P but with the addition of glycerol. The fosfomycin MIC for the single *uhpT* and the double-gene mutant for both transporters (Δ*glpT-uhpT*) were >64 mg/L for all the assayed conditions. For the remaining mutant strains, the MIC ranged from 1–4 mg/L with the addition of G6P, ≥32 mg/L in the absence of G6P, regardless of the addition of glycerol, except the mutant for Δ*glpR* gene (2 and 4 mg/L). 

Regarding the results observed by the agar dilution method, the addition of glycerol did not improve fosfomycin activity (MIC drop of ≥2 log_2_ dilution) with respect to fosfomycin alone or in combination with G6P.

Regarding the disk diffusion assays, considering the main inhibition zone, the results showed that the wild-type strains significantly increased their fosfomycin susceptibility with the addition of glycerol. This increase occurred with and without the addition of G6P for both wild-type strains, except for the *E. coli* ATCC25922 strain with glycerol (0.45 mg/mL) with G6P. 

The use of fosfomycin without G6P generated a reduced inhibition zone for the *E. coli* ATCC25922 but not for the *E. coli* MG1655 strains. It should be noted that the addition of glycerol also generated this reduced inhibition zone under certain conditions. The addition of glycerol did not modify the size of the reduced inhibition zone for the *E. coli* ATCC25922 strain but produced a reduced inhibition zone with the addition of G6P, also observed for the *E. coli* MG1655. 

With respect to the ∆*glpT* mutant, a significant increase in susceptibility was observed with the addition of 1.8 and 7 mg/mL of glycerol. This increase did not occur with the addition of G6P. Regarding the reduced inhibition zone, no changes were observed with the addition of the different concentrations of glycerol, with or without G6P.

The *E. coli* MG1655 ∆*uhpT* mutant showed, in the reduced inhibition zone, a significant increase in susceptibility with 1.8 and 7 mg/mL of glycerol, with and without G6P. Furthermore, the addition of any concentration of glycerol significantly increased the main inhibition zone, with or without G6P. 

*E. coli* MG1655∆*cyaA* strain showed a significant increase in susceptibility with 0.45 and 1.8 mg/mL of glycerol. The addition of 7 mg/mL of glycerol did not produce any effect, regardless of the addition of G6P. 

*E. coli* MG1655 ∆*glpT-uhpT,* ∆*glpR,* and ∆*glpK* strains did not show any significant increase in susceptibility in any of the conditions evaluated.

### 2.3. Fosfomycin and Glycerol Interaction Assay

The result of the interaction assay is shown in Table 2 and Figure S2. The combination of fosfomycin and glycerol showed synergistic activity (ZIP score > +10) G6P for the wild-type strains (*E. coli* ATCC25922: 16.3 ± 1.2 and *E. coli* MG1655: 16.5 ± 1.9), and for *E. coli* MG1655 ∆*uhpT* strain with G6P (12.3 ± 3.6) and without (11.3 ± 2.9). The most synergistic area occurred between 7–28 mg/mL of glycerol and 0.5–2 mg/L of fosfomycin for the *E. coli* ATCC25922 strain. Similar results were observed for the *E. coli* MG1655 strain, but at 0.25–1 mg/L of fosfomycin. In the case of *E. coli* MG1655 ∆*uhpT*, the greatest synergy was observed at the same concentration as that observed for the *E. coli* ATCC25922 strain with and without G6P.

Antagonism (ZIP score < −10) was observed against the *E. coli* MG1655 ∆*glpT-uhpT* strain under any condition (−12.8 ± 5.4 and −21 ± 3.8). The addition of glycerol did not interact with the rest of the evaluated strains.

### 2.4. Fosfomycin and Glycerol Time-Kill Assays

**Figure 4:**
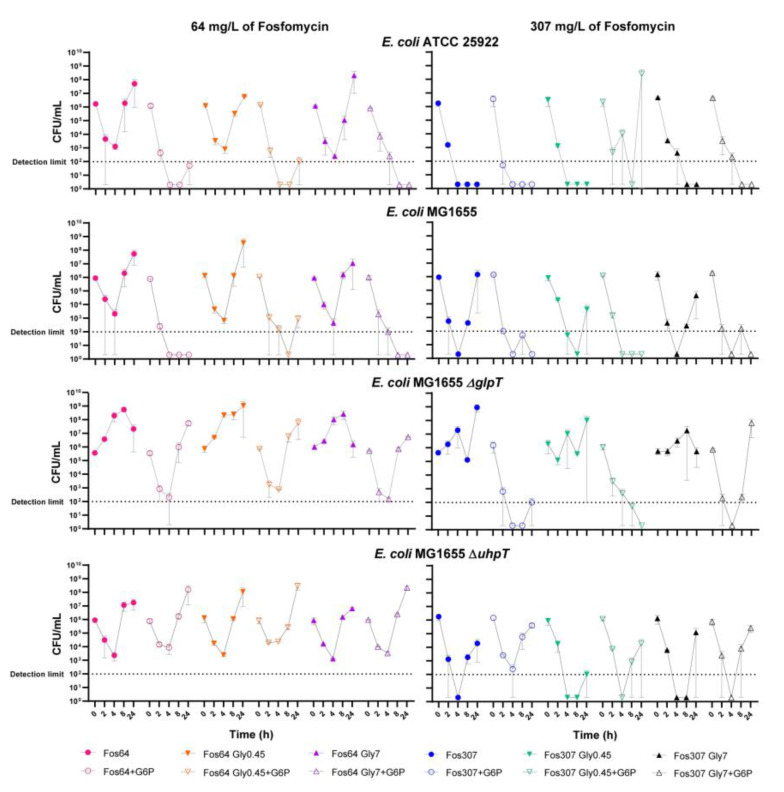
Time-kill assays of fosfomycin alone and in combination with glycerol (0.45 and 7 mg/L) and/or glucose-6-phosphate (G6P, 25 mg/L) against *E. coli* wild-type *E. coli* ATCC25922 and *E. coli* MG1655 wild-type and mutant derivative strains (∆*glpT* and ∆*uhpT*), at 0, 2, 4, 8, and 24 h. Bacterial concentrations (CFU/mL) are represented as symbols for mean and range.

*E. coli E. coli* ATCC25922, *E. coli* MG1655, and their mutant derivatives were evaluated at two concentrations of fosfomycin (64 and 307 mg/L), without glycerol, and with two concentrations (0.45 and 7 mg/mL). The results are shown in Figure 4 and Figure S3 (for *E. coli* ∆*glpT-uhpT,* ∆*cyaA,* ∆*glpR, and* ∆*glpK strains*). 

The addition of glycerol and G6P did not modify growth under control conditions (without fosfomycin, data not shown). Fosfomycin alone at 64 mg/L showed a bactericidal effect (decreased the bacterial burden ≥3 log_10_ CFU/mL) within the first 4 h against the wild-type and the *E. coli* MG1655 ∆*uhpT* mutant strains, but bacterial regrowths were observed at 8 and 24 h. The addition of G6P also showed a bactericidal effect within the first 4–8 h in all strains except *E. coli* MG1655 ∆*uhpT* and ∆*glpT-uhpT* and prevented the bacterial regrowth for the wild-type strains and the *E. coli* MG1655 ∆*glpR* and ∆*glpK* mutant strains. The addition of glycerol at both tested concentrations did not improve the bactericidal effect against any of the evaluated strains.

Except for *E. coli* MG1655 ∆*glpT* and ∆*glpT-uhpT* strains*,* fosfomycin alone at 307 mg/L showed a bactericidal effect against all the strains within the first hours. However, bacterial regrowth was observed except for the wild-type *E. coli* ATCC and MG1655 ∆*glpR* strains. The addition of G6P improved the bactericidal effect and prevented the regrowth in all the strains except for *E. coli* MG1655 ∆*uhpT* and ∆*glpT-uhpT.* The addition of glycerol at both concentrations did not improve the bactericidal effect in any of the evaluated strains. Only against *E. coli* MG1655 ∆*uhpT* strain fosfomycin combined with 0.45 mg/mL of glycerol showed an initial improved activity; however, bacterial regrowth was observed after 24 h.

## 3. Discussion

The present study evaluates the role of glycerol in enhancing fosfomycin activity against wild-type strains of *E. coli* and strains that harbor specific determinants of resistance to fosfomycin.

In general, the activation of the GlpT transporter using glycerol as an internal activator showed contradictory results on the activity of fosfomycin. 

The present study shows that glycerol, at physiological concentrations, can activate the *glpT* promoter, increasing *glpT* expression [13,16]. This result agrees with previous works showing the activation of genes (*glpACB*, *glpD glpFKX, glpTQ*, etc.) involved in the glycerol catabolism and regulated by the *glpR* repressor in *E. coli*, *P. aeruginosa* or *P. putida* [14,15,17]. However, this glycerol induction of *glpT* shows a delayed initiation in contrast to the rapid activation of the hexoses-6-phosphate transporter (*uhpT*) with the addition of G6P. These effects have been observed using GFP promoter fusions in real-time fluorescence monitoring and flow cytometry assays for *glpT* and *uhpT* genes. 

A possible explanation for this behavior was given by Nikel et al. [14], who observed a protracted lag phase in cultures of *P. putida* KT2440 growing in glycerol. 

The regulatory network of the *glp* genes needs the product of the first biochemical reaction (sn-glycerol-3-P) to derepress gene expression, which is otherwise inhibited by GlpR. However, the genes that encode the glycerol transporter and the kinase that produces G3P from glycerol are repressed by the GlpR protein. Thus, to get the transcription started is the low-probability effector-independent stochastic lifting of the repression. While the derepression process is taking place, this transcriptional architecture translates into different levels of metabolic activity (representing, in this context, the ability of the cells to catabolize glycerol). It is important to note that while the *glpT* gene is absent in *P. putida,* the regulation network is conserved in *E. coli* and *P. aeruginosa*. [1,15,18]

With regard to fosfomycin activity with the addition of glycerol, the present study shows distinct results depending on the characteristics of the assay.

In the disk diffusion assays, secondary and reduced inhibition zones were observed, suggesting the presence of subpopulations with different fosfomycin susceptibility, even with the use of glycerol and G6P. The maximum fosfomycin activity was observed against the *glpR* repressor mutant because the *glpT* transporter is fully derepressed and insensitive to the addition of glycerol. Additionally, the addition of glycerol did not show any effect in *glpK* mutant due to the inability to transform glycerol into glycerol-3-phosphate [16].

However, fosfomycin activity was observed, similar to that observed against the *E. coli* MG1655 wild-type strain without glycerol, and this could be partially explained by the intracellular biosynthesis of sn-glycerol-3-phosphate as a precursor of phospholipid synthesis [19]. The absence of activity was observed in the double-gen mutant Δ*glpT-uhpT* strain, indicating that the activation of the fosfomycin transporters is the main control factor of fosfomycin activity, as previously observed by Ballesteros et al. [1].

It is also important to note that the reduced inhibition zones remained unchanged, irrespective of the glycerol concentration or the addition of G6P, suggesting the presence of baseline defects in the complex regulatory networks of the fosfomycin transporters *glpT* and *uhpT* [6,20].

In the present study, discrepancies between disk diffusion and agar dilution susceptibility assays have been observed. These results agree with our previous studies in which fosfomycin susceptibility showed inconsistent results between broth and agar dilutions and agar diffusion techniques in collections of clinical isolates and isogenic mutants related to fosfomycin resistance, including heteroresistant strains [1,21,22]. Although there were no differences in the MIC of fosfomycin when glycerol was added to the assay, it must be noted that lower bacterial densities were observed (data not shown), indicating a mild synergistic effect not measurable with the assay. Regarding the interaction assays between fosfomycin and glycerol, only the wild-type strains and *E. coli* MG1655 ∆*uhpT* mutant showed synergistic activity. However, the time-kill assays did not show sensitization with the combination of fosfomycin and glycerol. It is important to note that in the time-kill assays, divergent results were observed between the replicates for wild-type strains and for the *E. coli* MG1655 ∆*uhpT.* For these strains, one replicate of the time-kill assay showed a total bacterial clearance and the other a bacterial regrowth after 24 h, which partially agrees with the results observed in the interaction assay. This divergence found between these replicates could be explained considering the rapid bactericidal activity of fosfomycin in contrast to our previous results showing the delayed activation of the *glpT* transporter, increasing the probability of the emergence of fosfomycin-resistant subpopulations as commonly observed in previous studies [1,23].

In conclusion, glycerol showed increased expression of the fosfomycin transporter *glpT* and a synergistic effect with fosfomycin in the interaction and disk diffusion assays. However, this molecule does not appear to be a good candidate as an adjuvant to fosfomycin therapy since the delay in the induction of *glpT* allows the selection of resistant subpopulations. It would therefore be necessary to perform further in vitro and in vivo studies aimed at overcoming these problems.

## 4. Materials and Methods

### 4.1. Bacterial Strains

*E. coli* ATCC25922 and *E. coli* MG1655 were used as the reference strain, and six isogenic mutants from *E. coli* MG1655 mutants (∆*glpT,* ∆*uhpT,* ∆*glpT-uhpT,* ∆*cyaA,* ∆*glpR,* and ∆*glpK*) were used in the assays. Isogenic mutants were generated from the KEIO collection [24] using phage P1*vir* transduction (Coli Genetic Stock Center [CGSC], Yale University, New Haven, CT, USA) as previously described [25].

### 4.2. Activation Kinetic of Fosfomycin Transporters Promoters

The activity of the fosfomycin transporter promoters *glpT* and *uhpT* was evaluated by monitoring the fluorescence accumulation in *E. coli* ATCC25922 and *E. coli* MG1655 carrying pMS201-P*glpT*::*gfp*mut2 or pMS201-P*uhpT*::*gfp*mut2 reporters, as described by Zaslaver et al. [26]. Bacterial culture fluorescence was determined after transferring overnight cultures in MHB to 96-well plates (Nunclon Delta Surface, Thermo Scientific, Waltham, MA, USA) with 200µL of MHB per well. Starting bacterial concentrations were adjusted to 5 × 10^5^ CFU/mL. The assays were carried out in Mueller Hinton Broth II (MHB) alone or supplemented with 0.45 mg/mL, 1.8 mg/mL, and 7 mg/mL of glycerol with and without G6P (Sigma-Aldrich, Madrid, Spain). Low, medium, and high glycerol physiological concentrations were assayed in combination with fosfomycin. The low glycerol concentration of 0.45 mg/mL corresponded to a target effective concentration to reduce the intracranial pressure [12]. The high glycerol concentration of 7 mg/mL corresponded to steady-state serum concentrations in patients with normal hepatic and renal function following constant intravenous infusion of 0.87 g/kg/h [27]. Finally, the medium glycerol concentration of 1.8 mg/mL was selected, corresponding to an intermediate concentration between 0.45 and 7 mg/mL in a log_2_ dilution scale. Green fluorescence (excited at 485 nm and measured at 540 nm) and bacterial growth (measured at 595 nm) were monitored each hour for 24 h with an Infinite200 Pro plate reader (Tecan Group AG, Männedorf, Switzerland). The assays were performed in duplicate. The OD:fluorescence ratio of the promoterless construction (pMS201-Ø::*gfp*mut2) was used as background for all experiments under the different growth conditions. Fluorescence was normalized to the OD, and the background was subtracted. The results were compared with ANOVA and Tukey’s multiple comparison test.

### 4.3. Population Analysis of Fosfomycin Transporters Promoters Induction

Flow cytometry assays were conducted to assess the population distribution of *glpT* and *uhpT* expressions (pMS201-P*glpT*::*gfp*mut2 or pMS201-P*uhpT*::*gfp*mut2 reporters) in *E. coli* ATCC25922 and *E. coli* MG1655. Furthermore, a negative expression control (pMS201-Ø::*gfp*mut2) and positive expression control (pMS201-P*cyaA*::*gfp*mut2) were used as described. Overnight cultures were diluted in 20 mL adjusting a bacterial concentration of 5 × 10^5^ CFU/mL. Bacterial growths were carried out in MHB, MHB supplemented with 25 mg/L of G6P, and MHB supplemented with glycerol: 0.45 mg/mL, 1.8 mg/mL, and 7 mg/mL. A milliliter sample of each condition was taken, and fluorescence was monitored at 4 and 24 h. The Beckman Coulter FC500 cytometer (Beckman Coulter, United States) was used for size (FSC) and complexity (SSC) measurements to define the bacterial population. Green fluorescence was excited using a blue laser (488 nm) and measured at 530/30 nm. The promoterless construction (pMS201-Ø::*gfp*mut2) was used as a negative control, and pMS201-P*cyaA*::*gfp*mut2 was used as a positive control.

### 4.4. Susceptibility Testing

Fosfomycin MIC was performed using the reference method, the agar dilution assay, following EUCAST standards [28]. Mueller Hinton II agar (MHA) plates (Sigma-Aldrich, Madrid, Spain), with and without 25 mg/L of G6P, with and without 7 mg/mL of glycerol. The fosfomycin (Sigma-Aldrich) concentration ranged from 0.5 to 64 mg/L. Plates were dried and incubated for 20 h at 35 °C. The assays were performed in duplicate.

Fosfomycin susceptibility was also determined using the disk diffusion method using blank antimicrobial disks loaded with 200µg of fosfomycin alone or with 50µg of G6P, following EUCAST recommendations [28]. Additionally, fosfomycin activity was assayed by supplementing MHA plates with the addition of 0.45 mg/mL, 1.8 mg/mL, and 7 mg/mL of glycerol. The diameter of the main and any other reduced inhibition zone was measured. The addition of glycerol was compared with respect to the negative control (MHA with or without G6P). The results were compared with ANOVA and Tukey’s multiple comparison test. The assays were performed in triplicate.

### 4.5. Fosfomycin and Glycerol Interaction Assay

The interaction between fosfomycin and glycerol was studied using the checkerboard assay in duplicate. Briefly, the interaction assay was performed with an inoculum of 5 × 10^5^ CFU/mL in 96-well plates with a final volume of 200 µL per well. Assays were performed with MHB with and without 25 mg/L G6P. Fosfomycin concentrations ranged from 0.125 to 128 mg/L, and glycerol concentrations from 0.45 to28.8 mg/mL. Wells without fosfomycin or glycerol were used as single-drug assays or growth controls. Bacterial densities were quantified spectrophotometrically by measuring optical density at 595 nm using an Infinite200 Pro plate reader. Bacterial viability was calculated as the ratio of the final bacterial OD to the final bacterial OD of the control growth well (without drug). A four-parameter log-logistic model was fitted to the data to generate dose–response curves for fosfomycin and glycerol. The degree of drug synergy across the entire dose–response matrix was analyzed using the response surface model, zero interaction potential (ZIP) [29]. The ZIP model assumes that two noninteracting drugs are expected to incur minimal changes in their dose–response curves. A delta score was calculated to quantify the deviation from the expectation of ZIP for a given dose pair and used the average delta over a dose–response matrix as a summary interaction score for a drug combination. Model construction and synergy studies were performed with the Synergyfinder package for R [29]. A synergy score of <−10 was considered antagonistic, a range from −10 to 10 as additive, and >10 as synergistic.

### 4.6. Fosfomycin and Glycerol Time-Kill Assays

Time-kill assays were performed in duplicate using fosfomycin concentrations of 0 (as growth control), 64, and 307 mg/L with and without 25 mg/L G6P, with and without 0.45 and 7 mg/mL of glycerol. The activity of fosfomycin alone at concentrations of 64 mg/L (lowest concentrations of fosfomycin in the non-susceptible category according to EUCAST breakpoints) and at 307 mg/L (mean maximum plasma concentrations in humans at steady-state after a dose of fosfomycin 8 g/Q8h), respectively, was determined [28,30].

Briefly, isolated colonies overnight of each strain were used to prepare the pre-inoculum in MHB and incubated overnight with shaking at 37 °C. The starting inoculum was set at 5 × 10^5^ CFU/mL in a final volume of 20 mL, and bacterial cultures were incubated at 37 °C with shaking. The number of viable CFUs was determined at 0, 2, 4, 8, and 24 h by serial dilution, followed by plating on MH agar plates with or without 64 mg/L fosfomycin and 25 mg/L G6P. The number of colonies was counted after 24 h of incubation.

## Figures and Tables

**Table 1:** Fosfomycin MIC (mg/L) results by agar dilution without and with 7 mg/mL of glycerol and 25 mg/L of glucose-6-phosphate (G6P).

		Fosfomycin MIC (mg/L)			
		No Glycerol		Glycerol 7 mg/L	
		No G6P	G6P 25 mg/L	No G6P	G6P 25 mg/L
*E. coli* **ATCC25922**		>64	2	64	2
*E. coli* **MG1655**	**Wild-type**	64	2	32	2
	**Δ*glpT***	>64	2	>64	2
	**Δ*uhpT***	>64	>64	>64	>64
	**Δ*cyaA***	>64	4	>64	2
	**Δ*glpR***	4	1	2	1
	**Δ*glpK***	>64	4	32	4
	**Δ*glpT-uhpT***	>64	>64	>64	>64

**Table 2:** ZIP synergy scores for the combination of fosfomycin with glycerol, with and without glucose-6-phosphate (G6P). Green and red colors show synergistic and antagonistic results, respectively.

ZIP SYNERGY SCORE	ATCC25922	MG1655						
		Wild-Type	Δ*glpT*	Δ*uhpT*	Δ*glpT-uhpT*	Δ*cyaA*	Δ*glpR*	Δ*glpK*
**Fosfomycin** **+ Glycerol**	16.4 ± 1.2	16.5 ± 1.9	2.5 ± 4.8	11.3 ± 2.9	−12.8 ± 5.4	−0.7 ± 6.4	2.7 ± 1.6	1.7 ± 1.9
**Fosfomycin** **+ Glycerol** **+ G6P**	6.8 ± 1.9	5.8 ± 2.6	6.9 ± 4.3	12.4 ± 3.6	−21 ± 3.8	1.8 ± 6.6	6.1 ± 1.6	2.7 ± 3.7

## Data Availability

The datasets used and/or analyzed during the current study are available from the corresponding author upon reasonable request.

## Supplementary Materials

The following supporting information can be downloaded at: https://www.mdpi.com/article/10.3390/antibiotics11111612/s1, Figure S1: Assay of promoter activities in response to glycerol (GLY) and glucose-6-phosphate (G6P). Time-course quantification of GFP expression as a measure of induction of the *glpT* and *uhpT* genes in the ATCC25922 and MG1655 strains after 4, 12, and 24 h of exposure. The data were normalized to promoterless strains. Error bars represent standard deviations. Concentrations of glycerol (Gly) of 0.45, 1.8, and 7 mg/mL and glucose-6-phosphate (G6P) of 25 mg/L were used as inductors; Figure S2: Interaction assay of fosfomycin in combination with glycerol against *Escherichia coli* ATCC25922 and MG1655 strains, represented as heat maps. The red and green areas represent synergy and antagonism, respectively. The white rectangles show the maximum synergistic area. The concentration–response curves for fosfomycin and glycerol alone are found on the left side of each heatmap. Figure S3: Time-kill assays of fosfomycin alone and in combination with glycerol (0.45 and 7 mg/L) and/or glucose-6-phosphate (G6P, 25 mg/L) against *E. coli* wild-type *E. coli* MG1655 mutant derivative strains (∆*glpT-uhpT,* ∆*cyaA,* ∆*glpR,* and ∆*glpK*), at 0, 2, 4, 8, and 24 h. Bacterial concentrations (CFU/mL) are represented as symbols for mean and range.
