# Supplementary material for: Effect of Glycerol on Fosfomycin Activity against Escherichia coli
Source: Antibiotics (Basel). 2022 Nov 12;11(11):1612. doi: 10.3390/antibiotics11111612 (PMC9686493; doi:10.3390/antibiotics11111612)
Supplement: Supplementary file 1 [file antibiotics-11-01612-s001.zip › antibiotics-2014483-Supplementary.pdf]

Supplementary Materials:

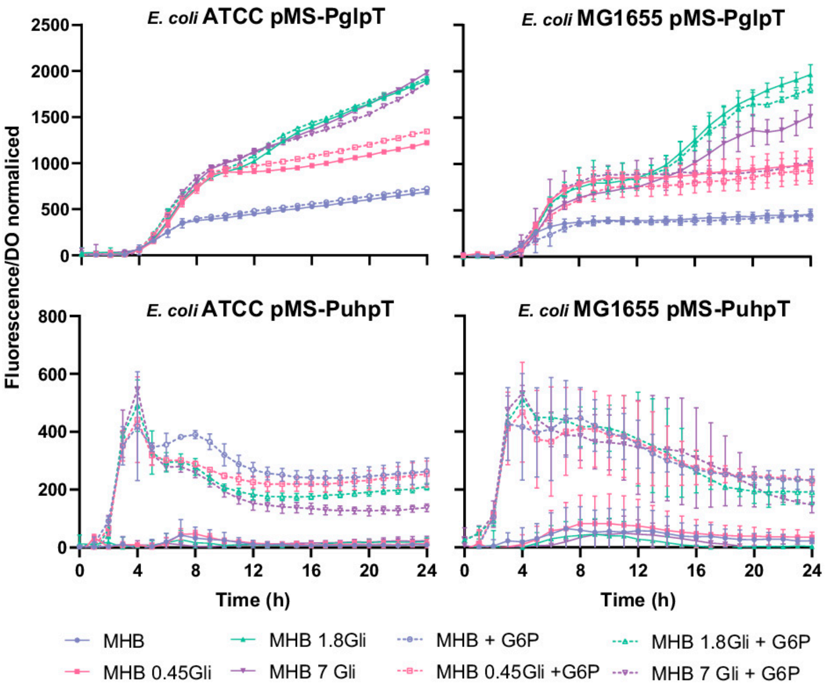

Figure S1. Assay of promoter activities in response to glycerol (GLY) and glucose-6-phosphate (G6P). Time-course quantification of GFP expression as a measure of induction of the *glpT* and *uhpT* genes in the ATCC25922 and MG1655 strains after 4, 12, and 24 h of exposure. The data were normalized to promoterless strains. Error bars represent standard deviations. Concentrations of glycerol (Gly) of 0.45, 1.8, and 7 mg/mL and glucose-6-phosphate (G6P) of 25 mg/L were used as inductors;.

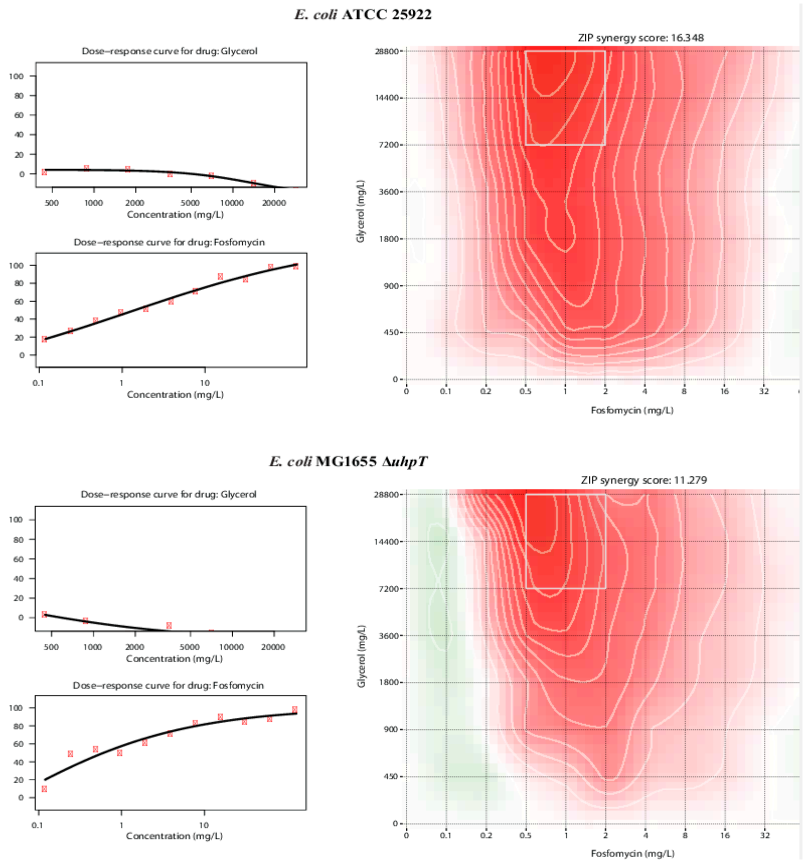

Figure S2. Interaction assay of fosfomycin in combination with glycerol against *Escherichia coli* ATCC25922 and MG1655 strains, represented as heat maps. The red and green areas represent synergy and antagonism, respectively. The white rectangles show the maximum synergistic area. The concentration–response curves for fosfomycin and glycerol alone are found on the left side of each heatmap.

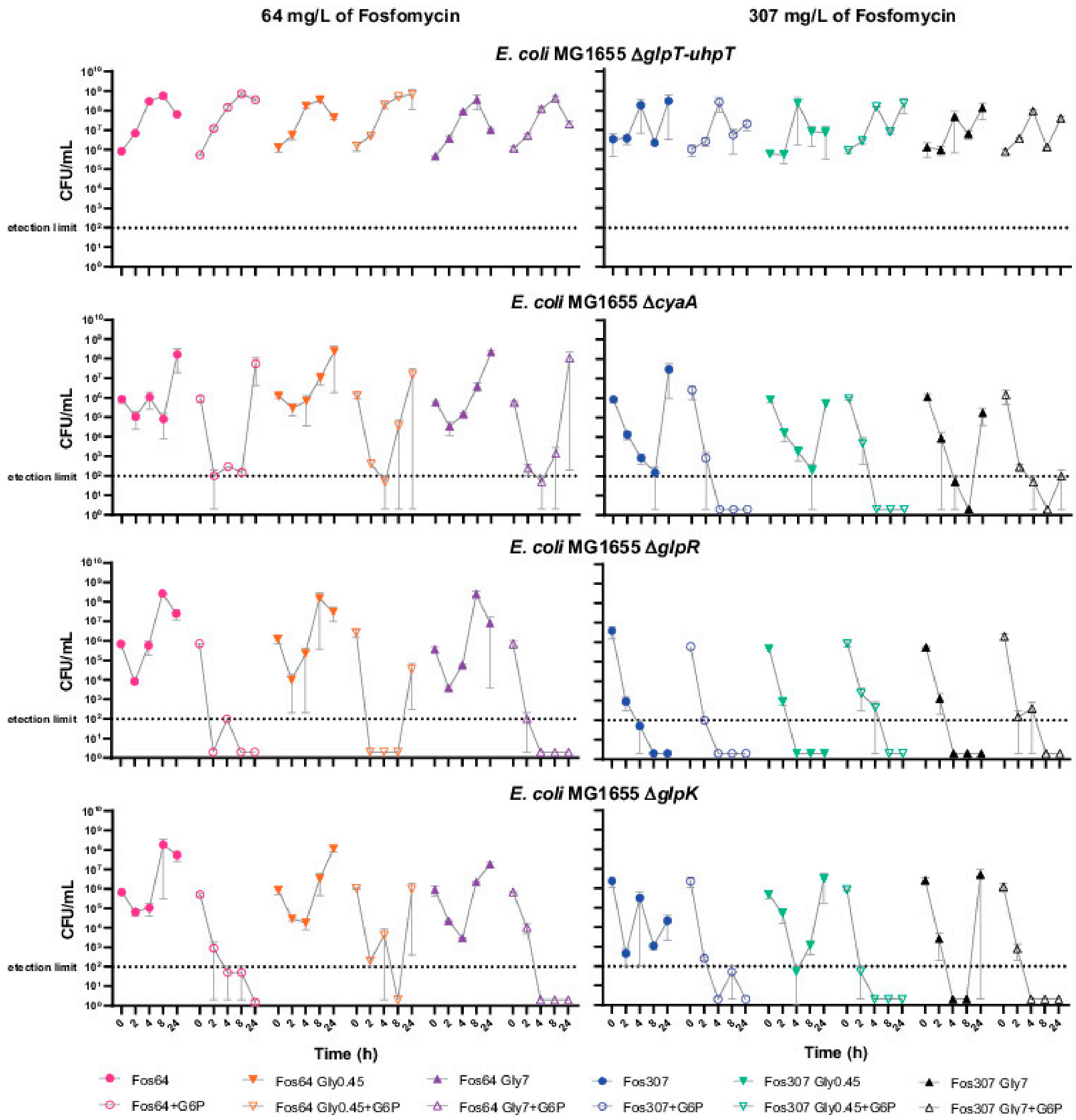

Figure S3. Time-kill assays of fosfomycin alone and in combination with glycerol (0.45 and 7 mg/L) and/or glucose-6-phosphate (G6P, 25 mg/L) against *E. coli* wild-type *E. coli* MG1655 mutant derivative strains (Δ*glpT-uhpT*, Δ*cyaA*, Δ*glpR*, and Δ*glpK*), at 0, 2, 4, 8, and 24 h. Bacterial concentrations (CFU/mL) are represented as symbols for mean and range.
